# Supplementary material for: Iranian primary healthcare system’s response to the COVID-19 pandemic using the healthcare incident command system
Source: PLoS One. 2023 Aug 22;18(8):e0290273. doi: 10.1371/journal.pone.0290273 (PMC10443878; doi:10.1371/journal.pone.0290273)
Supplement: S1 Table — (DOCX) [file pone.0290273.s001.docx]

| **S1 Table:** **Dimension scoring of the HICS evaluation tool.** | | | |
| --- | --- | --- | --- |
| **Dimension Name** | **low category scores (below the first quarter)** | **Medium category scores (within the quarters one to three)** | **High category scores (above the third quarter)** |
| Total Incident Management and Command | Score <72.22 | 72.22<Score>86.94 | Scores>86.94 |
| Organizational support | Score <80.00 | 80.00<Score>93.33 | Scores>93.33 |
| Operational | Score <66.66 | 66.66<Score>83.33 | Scores>83.33 |
| Coordination | Score <74.00 | 74.00<Score>96.00 | Scores>96.00 |
| Communication | Score <72.00 | 72.00<Score>90.00 | Scores>90.00 |
| Planning | Score <60.00 | 60.00<Score>80.00 | Scores>80.00 |
| Logistic | Score <58.00 | 58.00<Score>80.00 | Scores>80.00 |
| Human resource management | Score <80.00 | 80.00<Score>90.00 | Scores>90.00 |

**Iranian Primary Healthcare System's Response to the COVID-19 Pandemic using the Healthcare Incident Command System**
