# Supplementary material for: Iranian primary healthcare system’s response to the COVID-19 pandemic using the healthcare incident command system
Source: PLoS One. 2023 Aug 22;18(8):e0290273. doi: 10.1371/journal.pone.0290273 (PMC10443878; doi:10.1371/journal.pone.0290273)
Supplement: S2 File — (DOCX) [file pone.0290273.s003.docx]

**Iranian Primary Healthcare System's Response to the COVID-19 Pandemic using the Healthcare Incident Command System**

| **S2 File: Questionnaire to evaluate primary healthcare (PHC) response to the COVID-19 pandemic on the district health networks (DHNs) with inactive or no HICS** | | | | | |
| --- | --- | --- | --- | --- | --- |
|  | **Best** | **Good** | **Average** | **Bad** | **Worst** |
| 1- what was the executive capability of the head of PHC during the COVID-19 pandemic? |  |  |  |  |  |
| 2- To what extent have stakeholders, including partner organizations, support and relief groups, and other emergency response teams, been satisfied with the performance of the PHC in the COVID-19 pandemic in achieving the goals and objectives of the organization? |  |  |  |  |  |
| 3- what was the capacity of the head of PHC and his deputies to make emergency decisions during the COVID-19 pandemic? |  |  |  |  |  |
| 4- How much support logistics infrastructure such as vehicles, services, supplies were available during the COVID-19 pandemic? |  |  |  |  |  |
| 5- How do you evaluate the PHC response to the incident in the shortest possible time during the COVID-19 pandemic? |  |  |  |  |  |
| 6- How do you evaluate the PHC response to the incident based on the approved duties and protocols during the COVID-19 pandemic? |  |  |  |  |  |
| 7- To what extent was the PHC disaster management team able to implement their functions based on available resources during the COVID-19 pandemic? |  |  |  |  |  |
| 8- How do you evaluate the activation of the PHC headquarter units based on the approved rapid response plan during the COVID-19 pandemic? |  |  |  |  |  |
| 9- How do you evaluate the achievement of goals quantitatively and qualitatively according to EOC predictions during the COVID-19 pandemic? |  |  |  |  |  |
| 10- How do you evaluate the activation of the PHC management and operational units at different levels based on the approved protocol during the COVID-19 pandemic? |  |  |  |  |  |
| 11- Based on the localized trainings that were taught to the members before the onset of the COVID-19 crisis, how do you evaluate the activation process of the PHC headquarter units during the COVID-19 pandemic? |  |  |  |  |  |
| 12- In the preparedness phase, based on the instructions, how do you evaluate the PHC table-top maneuver (if the maneuver was performed; if not, do not answer the question)? |  |  |  |  |  |
| 13- In the preparedness phase, based on the instructions, how do you evaluate the PHC full-scale exercise (if the maneuver was performed; if not, do not answer the question)? |  |  |  |  |  |
| 14- How do you evaluate the PHC coordination with the relevant EOC in order to activate the management and operational units during the COVID-19 pandemic? |  |  |  |  |  |
| 15- How do you evaluate the PHC planning actions during the COVID-19 pandemic? |  |  |  |  |  |
| 16- How do you evaluate the PHC logistics and support functions during the COVID-19 pandemic? |  |  |  |  |  |
| 17- How do you evaluate the administrative and financial functions of PHC during the COVID-19 pandemic? |  |  |  |  |  |
| 18- How do you evaluate the operational functions of PHC during the COVID-19 pandemic? |  |  |  |  |  |
| 19- How do you evaluate the coordination between the head of PHC and the safety manager during the COVID-19 pandemic? |  |  |  |  |  |
| 20- How do you evaluate the coordination between the head of PHC and the security manager during the COVID-19 pandemic? |  |  |  |  |  |
| 21- How do you evaluate the coordination between the head of PHC and the public information manager during the COVID-19 pandemic? |  |  |  |  |  |
| 22- How do you evaluate the coordination between the head of PHC and the coordination and communication manager during the COVID-19 pandemic? |  |  |  |  |  |
| 23- How do you evaluate the coordination between the head of PHC, epidemiologists, infectious disease specialists as medical/ technical experts during the COVID-19 pandemic? |  |  |  |  |  |
| 24- How do you evaluate the coordination between the head of PHC and operation manager during the COVID-19 pandemic? |  |  |  |  |  |
| 25- How do you evaluate the coordination between the head of PHC and planning manager during the COVID-19 pandemic? |  |  |  |  |  |
| 26- How do you evaluate the coordination between the head of PHC and finance /administration manager during the COVID-19 pandemic? |  |  |  |  |  |
| 27- How do you evaluate the coordination between the head of PHC and logistic manager during the COVID-19 pandemic? |  |  |  |  |  |
| 28- How do you evaluate the communications and coordination of the PHC disaster management team regarding the reporting of actions during the COVID-19 pandemic? |  |  |  |  |  |
| 29- How do you evaluate the coordination of the head of PHC for reporting to the media during the COVID-19 pandemic? |  |  |  |  |  |
| 30- How do you evaluate the relationship between the PHC operations manager and the heads of the sub-branches (in the same section) during the COVID-19 pandemic? |  |  |  |  |  |
| 31- How do you evaluate the relationship between the PHC planning manager and the heads of the sub-branches (in the same section) during the COVID-19 pandemic? |  |  |  |  |  |
| 32- How do you evaluate the relationship between PHC logistic manager and the heads of the sub-branches (in the same section) during the COVID-19 pandemic? |  |  |  |  |  |
| 33- How do you evaluate the relationship between PHC administrative and financial manager and the heads of the sub-branches (in the same section) during the COVID-19 pandemic? |  |  |  |  |  |
| 34- How do you evaluate the communication between PHC disaster management members based on the common operational language during the COVID-19 pandemic? |  |  |  |  |  |
| 35- How do you evaluate the PHC performance changing based on EOC contingency plans, according to the type of incident, during the COVID-19 pandemic? |  |  |  |  |  |
| 36- How do you evaluate the PHC performance based on the Emergency Operation Plan book (National Disaster Response Plan in Iran) in the COVID-19 pandemic? |  |  |  |  |  |
| 37- How do you evaluate the announcement to deactivate and terminate PHC operations in coordination with the relevant EOC during the COVID-19 pandemic? |  |  |  |  |  |
| 38- How do you evaluate the sufficiency of equipment and resources available to PHC during the COVID-19 pandemic? |  |  |  |  |  |
| 39- How do you evaluate the PHC support for rapid response teams during the COVID-19 pandemic? |  |  |  |  |  |
| 40- How do you evaluate the availability of crisis communication equipment and devices such as wireless, telephone, satellite phone, and other items, during the COVID-19 pandemic? |  |  |  |  |  |
| 41- How do you evaluate the proportionality of operational support costs to the predicted expenses of the PHC during the COVID-19 pandemic? |  |  |  |  |  |
| 42- How do you evaluate the sufficiency of the number of members to implementing PHC operations during the COVID-19 pandemic? |  |  |  |  |  |
| 43- How do you evaluate the presence of PHC members in the COVID-19 crisis after their call to action in their roles? |  |  |  |  |  |
| 44- How do you evaluate the actions of PHC members to implementing processes, based on approved methods, during the COVID-19 crisis? |  |  |  |  |  |
| 45- How do you evaluate the speed of PHC members to implementing processes during the COVID-19 crisis? |  |  |  |  |  |
| 46- How do you evaluate determining a specific successor (at least one rank) for each of the PHC role and responsibility in the COVID-19 crisis |  |  |  |  |  |
| 47- How do you evaluate the presence of specific and defined communication methods for each PHC position and two categories of substitutes during the COVID-19 pandemic? |  |  |  |  |  |
| 48- How do you evaluate the proper placement of PHC members in their roles and responsibilities during the COVID-19 pandemic? |  |  |  |  |  |
| 49- How do you evaluate the implementation of the PHC process based on personnel safety during the COVID-19 crisis? |  |  |  |  |  |
| 50- How do you evaluate the level of compliance with the EOC regulations for the response phase by the PHC members during the COVID-19 crisis? |  |  |  |  |  |
