# Supplementary material for: Iranian primary healthcare system’s response to the COVID-19 pandemic using the healthcare incident command system
Source: PLoS One. 2023 Aug 22;18(8):e0290273. doi: 10.1371/journal.pone.0290273 (PMC10443878; doi:10.1371/journal.pone.0290273)
Supplement: Attachment — Submitted filename: Response to Reviewers. number3.docx [file pone.0290273.s004.docx]

PLOS ONE JOURNAL

Dear Editor

Thank you very much for considering our manuscript for publication. We appreciate the comments made by the respected the reviewers.

We carefully read the comments and considered the comments and made changes and /or made our manuscript clearer accordingly.

With respect to the opinions of the reviewers and editors, the article was revised. Please note that all changes are done in the main text.

**Journal Requirements:**

We've checked your submission and before we can proceed, we need you to address the following issues:

1. Thank you for stating the following financial disclosure:

[This study (no: IR.MUK.REC.1399.020.) was conducted with the financial support of the Disaster Risk Management Office in Deputy for Health at Ministry of Health and Medical Education (MOHE) in IRAN. The funding body did not have any other roles in the study design, collection, analysis, and interpretation of data, decision to publish and in writing the manuscript.]

At this time, please address the following queries:

a) Please clarify the sources of funding (financial or material support) for your study. List the grants or organizations that supported your study, including funding received from your institution.

b) State what role the funders took in the study. If the funders had no role in your study, please state: “The funders had no role in study design, data collection and analysis, decision to publish, or preparation of the manuscript.”

c) If any authors received a salary from any of your funders, please state which authors and which funders.

d) If you did not receive any funding for this study, please state: “The authors received no specific funding for this work.”

Please include your amended statements within your cover letter; we will change the online submission form on your behalf.

**Response:** Thanks for your comment. Based on your comment, based on ‘Financial Disclosure’, We revised and highlighted my amended statements (related to the funding section) in the **cover letter**, but we were unable to find the “Financial Disclosure” in the “additional information” section during the resubmission of the revised version, unfortunately this section was hidden. we hope this section will be added to the online version.
